# Is silence golden? Effects of auditory stimuli and their absence on adult hippocampal neurogenesis

**DOI:** 10.1007/s00429-013-0679-3

**Published:** 2013-12-01

**Authors:** Imke Kirste, Zeina Nicola, Golo Kronenberg, Tara L. Walker, Robert C. Liu, Gerd Kempermann

**Affiliations:** 1 CRTD, DFG Research Center for Regenerative Therapies Dresden, Fetscherstraße 105, 01307 Dresden, Germany; 2 Brain Imaging and Analysis Center (BIAC), Duke University Medical Center, Durham, NC 27710 USA; 3 Klinik und Poliklinik für Psychiatrie und Psychotherapie, Charité- Universitätsmedizin Berlin, Charité Campus Mitte, 10117 Berlin, Germany; 4 Department of Biology, Emory University, Atlanta, GA USA; 5 German Center for Neurodegenerative Diseases (DZNE) Dresden, Arnoldstraße 18b, 01307 Dresden, Germany

**Keywords:** Plasticity, Stem cells, Hippocampus, Mouse, Learning

## Abstract

We have previously hypothesized that the reason why physical activity increases precursor cell proliferation in adult neurogenesis is that movement serves as non-specific signal to evoke the alertness required to meet cognitive demands. Thereby a pool of immature neurons is generated that are potentially recruitable by subsequent cognitive stimuli. Along these lines, we here tested whether auditory stimuli might exert a similar non-specific effect on adult neurogenesis in mice. We used the standard noise level in the animal facility as baseline and compared this condition to white noise, pup calls, and silence. In addition, as patterned auditory stimulus without ethological relevance to mice we used piano music by Mozart (KV 448). All stimuli were transposed to the frequency range of C57BL/6 and hearing was objectified with acoustic evoked potentials. We found that except for white noise all stimuli, including silence, increased precursor cell proliferation (assessed 24 h after labeling with bromodeoxyuridine, BrdU). This could be explained by significant increases in BrdU-labeled Sox2-positive cells (type-1/2a). But after 7 days, only silence remained associated with increased numbers of BrdU-labeled cells. Compared to controls at this stage, exposure to silence had generated significantly increased numbers of BrdU/NeuN-labeled neurons. Our results indicate that the unnatural absence of auditory input as well as spectrotemporally rich albeit ethological irrelevant stimuli activate precursor cells—in the case of silence also leading to greater numbers of newborn immature neurons—whereas ambient and unstructured background auditory stimuli do not.

## Introduction

Adult neurogenesis adds plasticity to the dentate gyrus of the hippocampus and is involved in key functions such as pattern separation (Aimone et al. 2010; Clelland et al. 2009) and avoidance of catastrophic interference (Appleby and Wiskott 2009; Wiskott et al. 2006) by adding flexibility to the network in situations where novel information has to be integrated into established representations (Garthe et al. 2009; Dupret et al. 2008). Adult neurogenesis is regulated by behavioral activity. Both physical activity and exposure to a challenging environment increase adult neurogenesis but do so by different means (Kronenberg et al. 2003). Non-specific stimuli like physical activity enhance the proliferation of precursor cells and lead to an increased potential in form of a larger pool of “neuroblasts” and immature neurons that can be recruited in case of a cognitive challenge. In contrast, exposure to an enriched environment promotes the survival of newborn neurons. Accordingly, the two interventions turned out to be additive in their effect (Fabel et al. 2009).

The new immature neurons own particular functionality in that they are more likely to generate action potentials in response to incoming stimuli due to their particular balance between excitatory and inhibitory input (Marin-Burgin et al. 2012). The threshold for LTP induction is reduced in these neurons (Schmidt-Hieber et al. 2004; Snyder et al. 2001). In fact, the LTP that is measurable in the dentate gyrus under physiological conditions is contributed by the newborn neurons during this critical period of their development (Garthe et al. 2009; Saxe et al. 2006). Thus, the immature new neurons are assumed to be more easily excitable than older cells, biasing the input towards the more plastic subpopulation of cells (Marin-Burgin et al. 2012). The hypothesis is that this mechanism allows flexible adaptation and learning of new information in previously established contexts. Non-specific stimuli would increase precursor cell proliferation to increase a pool of cells that can be recruited if cognitive demand arises (for detailed discussion see: Fabel et al. 2009).

The finding that exercise would have this effect on proliferation raised the question, whether other non-specific stimuli would also lead to an increased availability of potentially recruitable cells. Presumably, the intrinsic stimulus during physical activity essentially consists of proprioception and vision. Likewise, there are numerous reports on links between the vestibular system and hippocampal function [(Brandt et al. 2005); see Ref. Smith et al. (2010) for review] even though effects on adult neurogenesis have not yet been specifically addressed. In order to identify relevant sensory stimuli independent of locomotion, we here focused on auditory input as a potential signal to affect adult hippocampal neurogenesis.

**Fig. 1:**
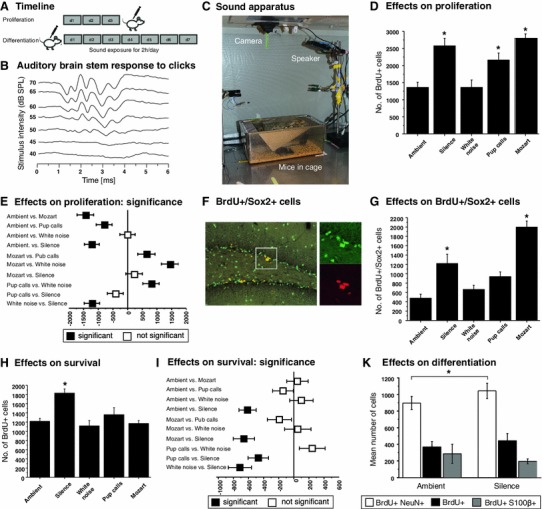
Regulation of adult hippocampal neurogenesis in dependency of auditory stimuli. **a** We used two different approaches to address both proliferation and survival/differentiation by injection of BrdU either 24 h before daily sounds exposure or after daily sound exposure (see “Materials and methods” for details). During the auditory stimulation for 2 h each day, animals were kept in anechoic sound isolation box to prevent outside interference (**c**). Auditory brain stem responses were measured in mice at the experimental age of 8 weeks to control for hearing abilities during the experiments and to adjust the dB levels (**b**). While white noise did not show any effect on the number of proliferating cells in the adult dentate gyrus, all other stimuli significantly increased the size of the population of BrdU+ cells (**d**, **e**, **a**). In case of exposure to silence as well as Mozart’s piano music (KV 448), we found particular increase in the population of BrdU-marked Sox2-positive precursor cells (**f**, **g**). The side length of the *box* in the large panel of (**f**) equals 150 μm. Differentiation, in contrast, was only significantly affected by silence (**h**, **k**) with a slight increase in the number of BrdU/NeuN double-positive new Neurons (**i**)

Noise trauma with inner ear hair cell loss has led to a reduction of precursor cell proliferation in the hippocampus of rats (Kraus et al. 2010). A potential positive regulatory effect of sound on the early steps of adult hippocampal neurogenesis, however, has not yet been explored. We asked how different types of auditory stimuli would affect the baseline regulation of adult hippocampal neurogenesis (Fig. 1a).

We used ambient noise in the animal facility (animal house noise) as baseline and exposed our mice to four different conditions: (1) white noise as unstructured auditory stimulus; (2) mouse pup calls as structured stimulus that is for mice common and relevant; (3) Mozart piano music as a structured stimulus, unknown and presumably irrelevant to mice; and (4) silence.

## Materials and methods

### Animals

All experiments were performed according to national and institutional guidelines and were approved by the Institutional Animal Care and Use Committee (IACUC) of Emory University.

Forty female C57BL/6J mice were obtained from The Jackson Laboratory (Bar Harbor, Maine, USA) and were 6 to 8 weeks old at the beginning of the experiments. For all experiments, mice were held under standard laboratory housing conditions with a 12-h light/dark cycle and ad libitum access to food and water. Delivered mice were given a minimum of 5 days for habituation after arrival and housed in groups of five animals per cage (*N* = 10 per group).

In comparison to other mouse strains, C57BL/6 animals have certain advantages in adult neurogenesis research because they typically show high neurogenic activity within the dentate gyrus, which responds readily to extrinsic stimuli (Kronenberg et al. 2003). We thus decided to use C57BL/6 despite the age-related presbyacusis that is typical for that strain (Hunter and Willott 1987).

### Auditory brainstem response

To control for potential hearing loss, 10 additional animals were used for measurement of the auditory brainstem response (ABR) in order to determine the average hearing ability of this strain at the given age of 8 weeks. This information was used to define the exposure parameters for the animals in the experiment.

Before testing, mice were anesthetized with 100 mg/kg body weight Ketamine and 0.3 mg/kg Medetomidine by intraperitoneal injection. During recording of the ABR, the eyes were covered with eye ointment to prevent dryness.

Once no more motor reflexes could be induced from the animal, silver wires were placed subdermally posterior to the stimulated ear, at the skull midline (ground) and at the contralateral bulla.

The recordings were done with a calibrated sound delivery system (Tucker-Davis Technologies, Gainesville, FL, USA) and BioSig program. Click trains and tones at 7, 32 or 65 kHz were presented to the anesthetized females at a sampling rate of 223,214.06 per second. All tones were presented with 3 ms duration, intensity in the range of 0 dB SPL to 75 dB SPL, and an onset/offset ramp of 1.5 ms. Auditory evoked activity was recorded, amplified (×10,000), and filtered (10 Hz–3 kHz). The hearing threshold was defined as the lowest intensity at which reliable responses were recorded.

At the end of the recordings, animals were injected subcutaneously with 0.05 ml lactated Ringer solution to prevent dehydration. The mice were monitored until they awoke and normal grooming and drinking behavior was observed.

### Exposure to sound stimuli

Ten animals of each group were placed into an anechoic sound isolation box (Acoustic Systems, Austin, TX, USA) for 2 h/day at the beginning of the dark phase of the light–dark cycle. Dependent on the experimental group, they were either exposed to standard animal house noise (group designated as “Ambient”), isolation from all sounds (“Silence”), white noise (“White noise”, with a bandwidth of 4–80 kHz), previously recorded pup calls (“Pup calls”) or Mozart’s Sonata for two pianos in D major, KV 448 (“Mozart”). The music was presented with a sample rate of 97,656.25 per second. The other sounds were presented at a sampling rate of 223,214.06 per second. The Mozart piece was transposed into the hearing range of C57BL/6J mice. First, a low pass filter was set to 1,236 Hz and applied ten times to the music. The wav file was then transposed by five octaves. Afterwards a high-pass filter was set to 5,953 Hz and applied two times. Spectral analysis showed a peak at 10 kHz. More than 90 % of the power lay between 5 and 20 kHz. Spectral analysis of the pup calls showed an average frequency of 65 kHz; the pup calls had been collected and modified as described elsewhere (Liu et al. 2003). The white noise was applied at an intensity of 70 dB SPL. All other stimuli varied in intensity (70 ± 10 dB SPL).

### Immunohistochemistry

To assess effects of auditory stimuli on precursor cell proliferation, the animals were exposed to the stimulus 2 h/day for 3 consecutive days, followed by an intraperitoneal injection of 50 mg/kg bodyweight Bromodeoxyuridine (BrdU, Sigma) 24 h after the last exposure (Fig. 1a). Again 24 h later, mice were killed and the brain was removed.

To address net neurogenesis (survival and differentiation), animals were injected with BrdU (50 mg/kg) and exposed to auditory stimulus for seven consecutive days. Perfusion was performed 24 h after the last exposure. In this particular paradigm BrdU counts reflect early survival rates of newly generated cells.

For the collection of the brains, all animals were deeply anesthetized with a mixture of 10 % Ketamine (0.3 ml/20 g body weight) and 2 % Xylaxin (0.1 ml/20 g bodyweight) and perfused transcardially with 0.9 % NaCl followed by 4 % paraformaldehyde (PFA) in 0.1 M KPBS buffer, pH 7.4. Brains were dissected from the skull, postfixed in 4 % PFA at 4 °C for 24 h and then transferred into 30 % sucrose in 0.1 M phosphate buffer, pH 7.4, for dehydration until they had sunk. Brains were then cut on a dry ice-cooled copper block with a table top sliding microtome (Leica, Bensheim) into 40-μm-thick coronal sections. Slides were stored at 4 °C in cryoprotectant solution containing 25 % ethylene glycol, 25 % glycerol, 25 % glycerin and 0.05 M phosphate buffer.

All sections were stained free floating with all antibodies diluted in Tris-buffered saline (TBS), pH 7.4, containing 3 % donkey serum and 0.1 %Triton X-100. For BrdU-immunohistochemistry, one-in-six sections from each brain were transferred into TBS and washed briefly. Sections were pretreated with 0.6 % H_2_O_2_ to block endogenous tissue peroxidase. After rinsing in TBS, the DNA was denatured in 2 N HCl for 30 min at 37 °C. Afterwards the sections were rinsed in 0.1 M borate buffer, pH 8.5, and thoroughly washed in TBS. Brain slices were incubated with the primary antibody overnight at 4 °C. Primary and secondary antibodies were diluted in TBS supplemented with 0.1 % Triton X-100 and 3 % donkey serum (TBS-plus). As primary antibody we used rat anti-BrdU in a concentration of 1:500 (Harlan Seralab, Loughborough, UK). On the next day, the sections were rinsed in TBS and TBS-plus and incubated with the secondary antibody for 2 h at room temperature. As secondary antibody we used donkey anti-rat-biotin-SP (Dianova, Hamburg, Germany) at a concentration of 1:250. ABC reagent (Vectastain Elite, Vector Laboratories, Burlingame, CA, USA) was applied for 1 h at a concentration of 9 μl/ml. Diaminobenzidine (DAB) (Sigma, Munich, Germany) was used as a chromogen at a concentration of 0.25 mg/ml in TBS with 0.01 % H_2_O_2_ and 0.04 % nickel chloride. The stained sections were thoroughly washed, incubated in Neoclear for 10 min and mounted with Neomount.

### Immunofluorescence

For immunofluorescence, 1-in-12 series of each brain were triple-labeled. The sections were transferred into TBS and washed briefly. The DNA was denatured in 2 N HCl for 30 min at 37 °C. Sections were then rinsed in 0.1 M borate buffer, pH 8.5, and thoroughly washed in TBS and TBS-plus. The brain sections were incubated with the primary antibodies overnight at 4 °C. Primary and secondary antibodies were diluted in TBS supplemented with 0.1 % Triton X-100 and 3 % donkey serum (TBS-plus). The next day, sections were rinsed in TBS and TBS-plus and incubated with secondary antibodies for 4 h at room temperature in the dark. Sections were then washed with TBS and coversliped in polyvinyl alcohol with diazabicyclooctane (DABCO) as anti-fading agent.

The primary antibodies were applied in the following concentrations: anti-BrdU (rat, 1:500; Harlan Seralab), anti-doublecortin (goat, 1:200; Santa Cruz Biotechnologies), anti-SOX2 (rabbit, 1:400, Chemicon, Temecula, USA), anti-NeuN (mouse, 1:100; Chemicon, Temecula, USA), anti-S100beta (rabbit, 1:2,500, Swant).

For secondary antibodies anti-rat rhodamine-X, anti-rabbit fluorescein isothiocyanate (FITC), anti-rabbit Cy5, anti-mouse FITC, anti-mouse Cy5, anti-goat Cy5 (all 1:250; Jackson Immunoresearch, West Grove, USA; distributor: Dianova, Hamburg, Germany) were used.

### Quantification and imaging

Quantification of cells labeled with DAB were determined using light microscopy on sections 240 μm apart for BrdU covering the entire hippocampus in its rostrocaudal extension as described previously (Kronenberg et al. 2003). Briefly, cells located in the granule cell layer and adjacent subgranular zone, defined as a two-cell bodies-wide zone of the hilus along the base of the granule cell layer were counted. Cells in the uppermost focal plane were excluded to avoid oversampling. For light microscopical analyses, a Leica CTR 6000 Microscope was used.

Phenotypic analyses of BrdU-positive cells in the stated differentiation stages were performed using multiple-stained series on sections 480 μm apart, also covering the entire hippocampus, using a spectral confocal microscope (TCS SP2 and TCS SP5; Leica, Nussloch, Germany). Appropriate gain and black level settings were determined on control slices stained with secondary antibodies alone. All images were taken in sequential scanning mode and further processed in Adobe Photoshop 7.0 and CS3. Only general contrast adaptations were made and images were not otherwise manipulated.

### Statistical analysis

Statistical analysis was performed either with Origin8 for Windows. Factorial analysis of variance (ANOVA) was performed for all comparisons of morphological data. Two-way ANOVA was followed by Fisher’s post hoc test, where appropriate. Differences were considered statistically significant at *p* < 0.05. All graphs are displayed as mean ± standard error of the mean (SEM).

## Results

With increasing age C57BL/6 mice undergo severe progressive sensorineural hearing loss (presbyacusis) starting around 4–5 months of age (Hunter and Willott 1987). To ensure the audibility of the auditory stimuli to our 2-month-old mice, we measured the auditory brainstem response (acoustic evoked potentials) to click sounds in C57BL/6 mice (Fig. 1b). This verified that the auditory stimuli that we intended to use in exposure would be above threshold for C57BL/6 at this age. Clicks equivalent to all of our stimuli are able to drive neural responses at the distinct frequency with the applied 70 dB SPL. Ambient baseline noise in the animal facility (Ambient) served as physiological baseline for our study.

Animals were exposed to the specific stimuli for 2 h per day for 3 days (Fig. 1a, c) in an anechoic chamber. One day after labeling cells with BrdU, White noise did not result in changing numbers of BrdU-positive cells, but all other stimuli increased proliferation (ANOVA: *F* (4.39) = 12.17, *p* = 1.62 × 10^−6^, Fig. 1d, e). The greatest numbers of labeled cells were seen for Silence and Mozart. When we broke down these numbers according to the expression of Sox2 as marker for early progenitor cell stages (type-1/2a, Fig. 1f), we saw that this increase was largely explained at this developmental stage (Fig. 1g). The stimuli indeed increased precursor cell proliferation.

At 7 days after division, the subset of cells that are destined for neuronal differentiation have exited from the cell cycle and only the minor fraction of label-retaining precursor cells remained proliferative (Encinas et al. 2011; Kempermann et al. 2003). The following period coincides with the electrophysiologically critical time window. We found that at this stage only the Silence group showed increased numbers of BrdU-positive cells, whereas all other groups were indistinguishable from the Ambient controls (ANOVA: *F* (4.42) = 10.73, *p* = 1.41251 × 10^−6^ Fig. 1h, i). The Silence and Ambient groups were further analyzed for the phenotype of the newborn cells. As known from other experiments, approximately two-thirds of these cells were neurons (based on NeuN expression) and around 15 % were S100β-positive astrocytes. The proportion of neurons was increased to 62 % in the silence condition from 57 % in the Ambient noise group and the difference barely missed conventional statistical significance (*p* = 0.056). In absolute terms, Silence resulted in statistically increased levels of neurogenesis (BrdU/NeuN-double-positive cells) at this stage (*p* = 0.008, Fig. 1k).

## Discussion

The present study shows that auditory stimuli can induce a response at the level of adult neurogenesis compared to normal baseline ambient noise or unstructured white noise. More interestingly, however, we found that silence and ethologically irrelevant sounds (Mozart) showed a stronger on proliferation effect than presumably natural and relevant sounds like pup calls. Both Mozart and Silence also represent highly novel stimuli, in line with the idea that adult hippocampal neurogenesis plays a role in the integration of novel information into pre-existing contexts.

To our initial surprise, silence, i.e., the complete absence of auditory input, was the only stimulus that elicited a strong response at the level of immature (7 day old) new neurons. But of the tested paradigms, silence might be the most arousing, because it is highly atypical under wild conditions and must thus be perceived as alerting. Functional imaging studies indicate that trying to hear in silence activates the auditory cortex, putting “the sound of silence”, the absence of expected sound, at the same level with actual sounds (Kraemer et al. 2005; Voisin et al. 2006). The alert elicited by such unnatural silence might stimulate neurogenesis as preparation for future cognitive challenges.

As in our previous set of experiments, one could now call for a combination of the non-specific and the specific stimulus in order to see whether a learning stimulus can recruit new neurons from the pool of new neurons generated in response to exposure to silence, as was the case for physical activity (Fabel et al. 2009). Given the small effect size here and challenges to the experimental design because of the different temporal properties of the two stimuli, this experiment is not trivial and, if done, should possibly be included into larger-scale studies on the general mechanisms underlying such two-step regulation. Based on the available data, we would predict that silence-activated progenitor cells should be usable for the response to cognitive challenges, because these new neurons have survived the initial wave of cell death after cell birth in the adult hippocampus (Kempermann et al. 2003) and are in a time window or critical period, during which the survival-promoting forces are effective (Tashiro et al. 2007; Kee et al. 2007; Gould et al. 1999).

Loud white noise can be a strong stressor (Lai 1987; Cheng et al. 2011). In our study, we purposefully used white noise that did not reach this damaging level. Our data did not reveal any substantial difference between ambient animal house noise and white noise. The white noise condition thus also serves as control for the anechoic chamber in which the animals were exposed to the sounds.

In any case, the role of stress in the regulation of adult hippocampal neurogenesis is complex. While strong acute social stress downregulates adult neurogenesis, the picture is less clear in other situations (Lucassen et al. 2010). The experience of sudden silence will represent a stressor, but so does the exposure to a running wheel (Van Praag et al. 1999). So the presence of stress per se is not incompatible with an increase in adult neurogenesis and the activation that is required for the neurogenic response might even be considered as “good stress” or eustress.

While few humans will agree that Mozart is “ethologically irrelevant noise,” for mice it should be. We have chosen Mozart’s Sonata for two pianos (KV 448) somewhat tongue-in-cheek, because it has received a notorious reputation in the context of the so-called “Mozart effect”, the controversial claim that listening to this sonata was sufficient to elicit improved learning in humans (Fudin and Lembessis 2004; Jenkins 2001). One study claimed that the effect on humans could be replicated in rats (Rauscher et al. 1998) and one report showed improved T-maze learning in mice after exposure to Mozart but not Beethoven (Aoun et al. 2005). The “Mozart effect” is largely discredited, also due to a rather unprecedented financial exploitation, even though there are actually interesting observations related to this paradigm, including reproducible studies on reduced epileptiform changes in the EEG (Lin et al. 2011) or on energy expenditure in preterm infants (Lubetzky et al. 2010). Such results are usually explained through an arousal elicited by the music (Lubetzky et al. 2010; Steele 2000). In this view, the Mozart piece KV 448 (first movement) would be nothing more but a particularly effective way to elicit a quite generic response. What is characteristic about the particular piece, however, is that it is highly patterned and has a rather fast beat. It represents an auditory stimulus that is not natural to mice and does not convey ethologically relevant information about the world. Nevertheless, its patterned nature might still resonate with sensory and cognitive mechanisms available to the mouse (compare, for example: (Schneider et al. 2010)). It would represent a stimulus of novelty and might thus also affect neurogenesis. We found that exposure to Mozart indeed induced an arousal-like effect at the precursor cell level. Unlike the complete absence of auditory signals, however, this increase in proliferating Sox2-positive cells was not followed by an increased availability of immature neurons. Why that might be, we can only speculate, but the important conclusion here is that regulation of neurogenesis obviously responds differentially to different auditory stimuli.

Adult neurogenesis has been hypothesized as crucial for flexible adaptations to environmental changes and thus displays an evolutionary advantage (Kempermann 2012). Modulation of the dentate gyrus cytoarchitecture caused by auditory stimuli is a particular case of experiencing the outside world, to which the animal has to respond. Our findings of different neurogenic responses to sounds be they structured or unstructured, relevant or irrelevant, and even present or absent, might reflect the preparation for novel information and behavioral contingencies, and thus increase the ability to adapt to environmental changes. Indeed, as acoustic stimuli (or possibly their absence) gain meaning to an animal through experience, auditory cortical plasticity occurs that may functionally improve the processing of those sounds, as has been demonstrated for pup calls (Liu and Schreiner 2007; Galindo-Leon et al. 2009).

Studies on auditory-dependent learning have also revealed changes in hippocampal activation (McIntosh and Gonzalez-Lima 1998; Grasby et al. 1993), and the conscious perception of sounds appears to activate the hippocampus (Laureys et al. 2000). These therefore support the idea that the hippocampus is also a site of higher order integration of sensory inputs (Braak et al. 1996; Jones and Powell 1970).
